# Possible involvement of Syndecan-1 in the state of COVID-19 related to endothelial injury

**DOI:** 10.1186/s12959-021-00258-x

**Published:** 2021-01-27

**Authors:** Keiko Suzuki, Hideshi Okada, Hiroyuki Tomita, Kazuyuki Sumi, Yoshinori Kakino, Ryu Yasuda, Yuichiro Kitagawa, Tetsuya Fukuta, Takahito Miyake, Shozo Yoshida, Akio Suzuki, Shinji Ogura

**Affiliations:** 1 Department of Pharmacy, Gifu University Hospital, Gifu, Japan; 2 Department of Emergency and Disaster Medicine, Gifu University Graduate School of Medicine, 1-1 Yanagido, Gifu, 501-1194 Japan; 3 Department of Tumor Pathology, Gifu University Graduate School of Medicine, Gifu, Japan

**Keywords:** COVID-19, Syndecan-1, Endothelial glycocalyx, D-dimer

## Abstract

**Background:**

The coronavirus infection 2019 (COVID-19) is associated with microvascular endothelial injury. Here, we report that syndecan-1, a component of endothelial glycocalyx, may reflect the disease state of COVID-19 related to endothelial injury.

**Case presentation:**

A patient with COVID-19 was transferred to the intensive care unit of our hospital. Computed tomography of the chest showed bilateral ground glass opacities, which was diagnosed as acute respiratory syndrome. The PaO_2_/F_I_O_2_ ratio gradually increased from 158 on hospitalization to 300 on Day 11, on which day the ventilator was withdrawn. However, serum syndecan-1 (SDC-1) level gradually decreased from 400.5 ng/ml at hospitalization to 165.1 ng/ml on Day 5. On Day 6, serum SDC-1 level increased to 612.9 ng/ml owing to a systemic thrombosis with an increase in D-dimer. Serum SDC-1 level then decreased until 206.0 ng/ml on Day 11 after a decrease in D-dimer. The patient was transferred to another hospital on Day 21 after hospitalization.

**Conclusions:**

In this case report, changes in serum SDC-1 level closely reflected the change in disease condition in a patient with COVID-19. Serum SDC-1 may be a useful biomarker for monitoring the disease state of critically ill patients with COVID-19.

## Background

Patients infected with the pandemic novel coronavirus (COVID-19) typically develop severe acute respiratory syndrome (ARDS). ARDS results from diffuse injury to cells that form the alveolar barrier, surfactant dysfunction, activation of innate immune response, and abnormal coagulation [1]. Recent reports have suggested that one of the mechanisms of ARDS induced by COVID-19 may be involved in microvascular endothelial cell injury [2]. Microvascular endothelial cell injury promotes thrombosis, particularly in the alveolar capillaries of COVID19 pneumonia patients [3, 4]. The glycocalyx, a gel like substance that coats the luminal surface of endothelial cells, largely inhibits platelet adhesion to the microvasculature [2]. Inflammation-induced degradation of the glycocalyx, which is measured by the glycocalyx biomarkers syndecan-1 (SDC-1), sP-selectin and hyaluronic acid, contribute to microvascular pathology in COVID-19 patients [5, 6]. Recently, the protection and restoration of glycocalyx has been suggested to be a key therapeutic target for COVID-19 [2, 5].

Here, we present the case of a COVID-19 patient with ARDS in whom serum SDC-1, a component of glycocalyx, was measured over time, and examine the association of SDC-1 as a reflection of the disease state of COVID-19 in relation to endothelial injury.

## Case presentation

The patient was a 63-year-old male with no medical history. He developed a 38 °C fever and visited his local general hospital. Reverse transcription polymerase chain reaction (RT-PCR) testing for severe acute respiratory syndrome coronavirus (SARS-CoV-2) nucleic acid in nasopharyngeal swabs was negative. The fever had not resolved 6 days after his initial symptoms and he visited the hospital again. Computed tomography (CT) of the chest showed bilateral ground glass opacities, and oxygen saturation (SpO_2_) under room conditions was 85%. Loop-Mediated isothermal amplification (LAMP) assay for SARS-CoV-2 nucleic acid in nasopharyngeal swabs was positive. On the same day, the patient was intubated and administered remdesivir (200 mg SID on Day 1, then 100 mg SID; d.i.v.), dexamethasone (6.6 mg SID; d.i.v.) and heparin sodium (10,000 units; d.i.v.). At 8 days after the initial symptoms, he was transferred to our hospital due to worsening respiratory status.

**Fig. 1:**
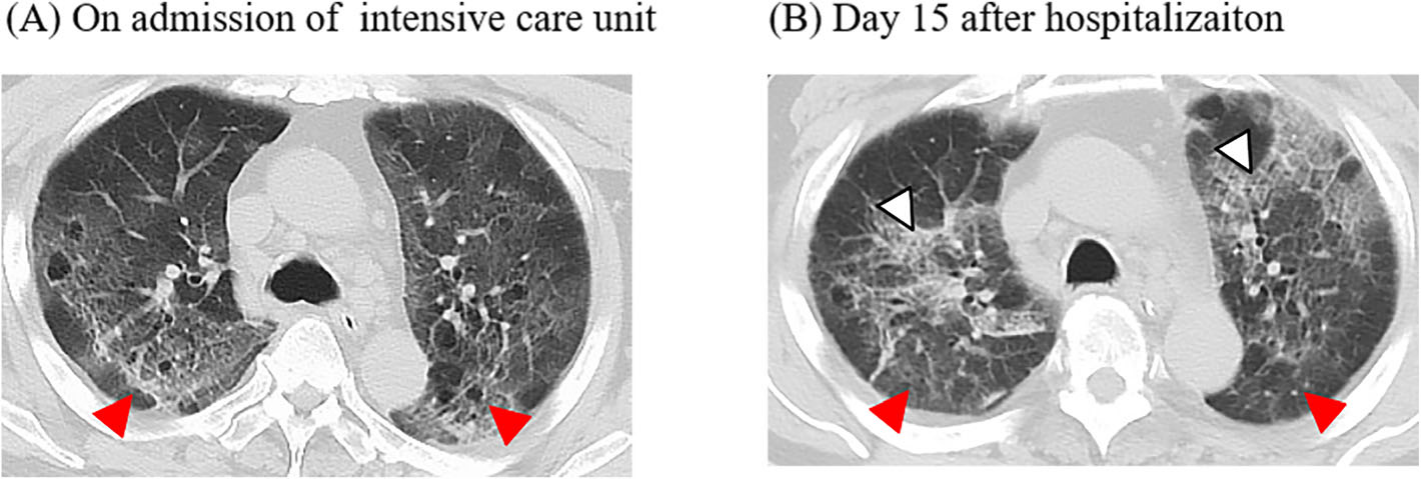
Computed tomography (CT) scan of the chest on admission to the intensive care unit (**a**) and on Day 15 after admission (**b**)

**Fig. 2:**
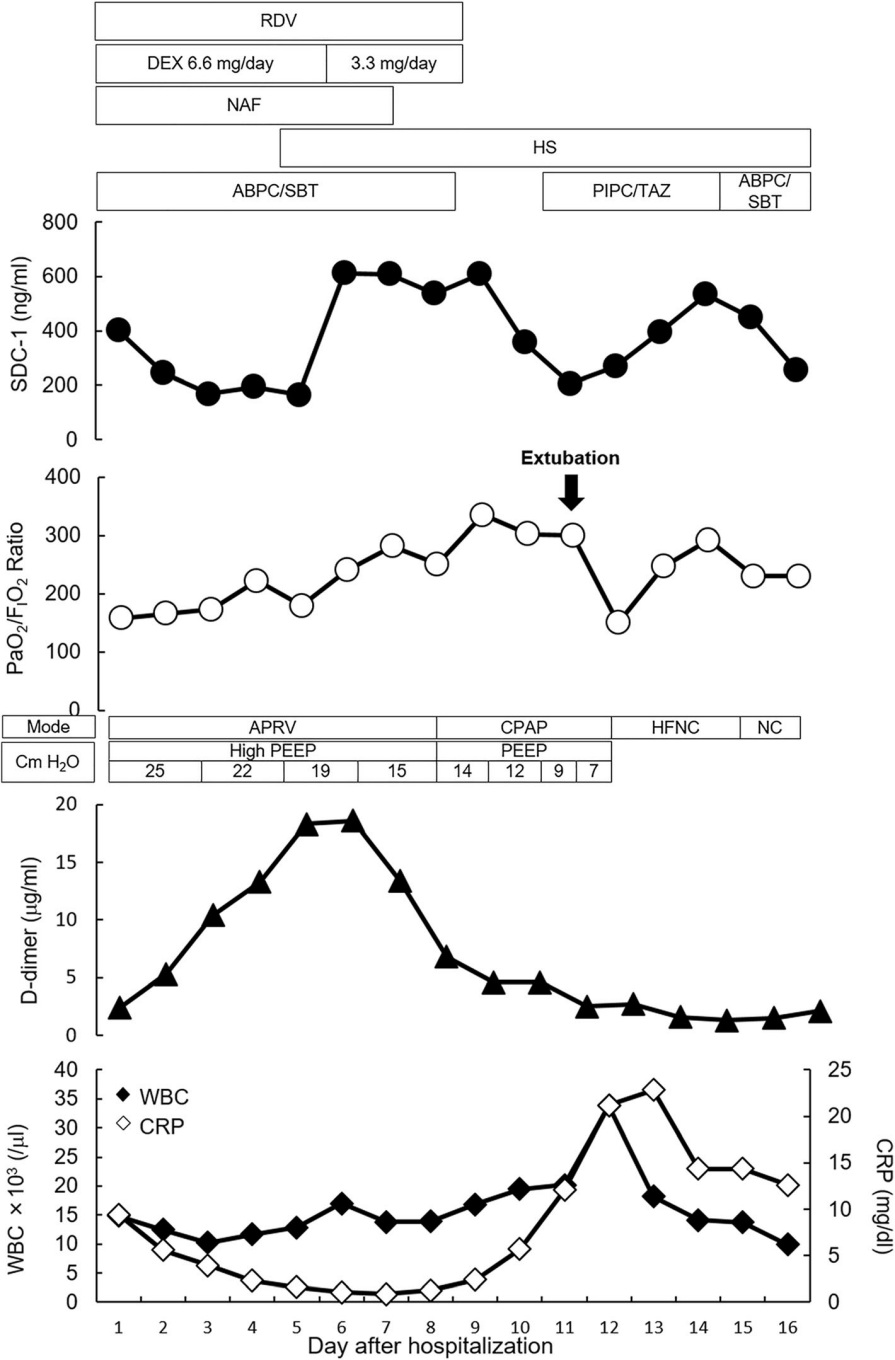
Time course of serum syndecan-1, respiratory function, and changes in levels of inflammatory and coagulation markers. RDV: Remdesivir, DEX: Dexamethasone, NAF: Nafamostat mesilate, HS: Heparin sodium, ABPC/SBT: Ampicillin/Sulbactam, PIPC/TAZ: Piperacillin/Tazobactam, APRV: Airway pressure release ventilation, CPAP: Continuous positive airway pressure, HNC: High **nasal** cannula, NC: **Nasal** cannula, PEEP: Positive end-expiratory pressure, SDC-1: Syndecan-1, PaO_2_/F_I_O_2_: Partial pressure of oxygen/Fraction of inspired oxygen, FDP: Fibrin degradation product, WBC: White blood cell, CRP: C-reactive protein

Laboratory test findings on admission to the intensive care unit (ICU) of our hospital were serum albumin, 2.4 g/dl; serum aspartate transaminase, 27 U/l; serum alanine aminotransferase, 27 U/l; serum creatinine, 0.74 mg/dl; serum creatine kinase, 33 U/l; white blood cells, 14.67× 10^3^ /μl; serum hemoglobin, 13.7 g/dl; platelets, 247× 10^3^ /μl; serum D-dimer, 2.4 μg/ml; and serum fibrinogen degradation products, 535 mg/dl. Arterial blood gas analysis on 0.6 fraction of inspired oxygen (F_I_O_2_) revealed a pH of 7.373, partial pressure of oxygen (PaO_2_) of 134 mmHg, partial pressure of carbon dioxide (PaCO_2_) of 34.9 mmHg, bicarbonate (HCO_3_^−^) of 19.8 mmol/l, base excess of − 4.3 mmol/l, and lactate of 10 mg/dl [Respirator mode: synchronous intermittent mandatory ventilation (SIMV), positive end-expiratory pressure (PEEP): 12 cmH_2_O]. CT of the chest showed bilateral ground glass opacities (Fig. 1a, red arrowhead), which was diagnosed as ARDS. Remdesivir (100 mg SID; d.i.v.) and dexamethasone (6.6 mg SID; d.i.v.) were continued, and nafamostat mesilate (6.25 mg/hr.; d.i.v.) and ampicillin/sulbactam (3 g q6hr; d.i.v.) were started. Serum syndecan-1 level, which was measured using an enzyme-linked immunosorbent assay (950.640.192; Diaclone, Besancon, Cedex, France), was 400.5 ng/ml (Fig. 2).

PaO_2_/F_I_O_2_ ratio gradually increased from the day after hospitalization, reaching 166 on Day 2, 173 on Day 3, 223 on Day 4 and 179 on Day 5. Similarly, serum SDC-1 level gradually decreased from day after hospitalization, reaching 244.6 on Day 2, 168.5 on Day 3, 194.0 on Day 4 and 165.1 ng/ml on Day 5. His dexamethasone dose was reduced to 3.3 mg on Day 6 (Fig. 2).

However, a daily increase in D-dimer was observed from the day after hospitalization, which was considered due to systemic thrombosis following endothelial cell injury by SARS-CoV-2. We re-administrated heparin sodium (15,000 units; d.i.v.) from Day 5. Levels peaked on Day 6 and gradually decreased thereafter (Fig. 2). Serum SDC-1 levels also increased again on Day 6 to 612.9 ng/ml, remained high until Day 8, then reduced after the decrease in D-dimer. On Day 7, nafamostat mesilate was discontinued. On Day 8, improvement in bilateral infiltrates was observed on chest X-ray (data not shown), and dexamethasone was discontinued. On Day 9, LAMP assay for SARS-CoV-2 nucleic acid in nasopharyngeal swabs was positive. On day 11, the patient was taken of the ventilator and switched to a high-flow nasal cannula because it was considered that the patient was not propagating SARS-CoV-2. Serum SDC-1 level at this time was decreased to 206.0 ng/ml.

Thereafter, although his PaO_2_/F_I_O2 ratio was temporarily decreased due to aspiration pneumonia and C-reactive protein and white blood cells were increased (Fig. 2), the patient improved with antibiotic treatment. Serum SDC-1 level increased to 533.6 ng/ml during treatment for aspiration pneumonia.

On Day 15, LAMP assay for SARS-CoV-2 nucleic acid in nasopharyngeal swabs was positive. CT of the chest on Day 15 showed an improvement of bilateral ground glass opacities compared with at hospitalization (Fig. 1b, red arrowhead), but other signs of pneumonia were seen (Fig. 1b, white arrowhead). The patient was transferred to another hospital on Day 21 after hospitalization.

## Discussion and conclusions

SDC-1, heparan sulfate proteoglycan, is a core protein of the glycocalyx whose degradation indicates endothelial injury [7–9]. Two recent studies reported serum SDC-1 levels in critically ill patients with COVID-19 [5,10]. Fraser et al. showed that critically ill patients with COVID-19 had higher serum SDC-1, in addition to sP-selectin and hyaluronic acid, particularly on Day 3 after admission to the ICU and thereafter [5]. Conversely, Hutchings et al. reported that serum SDC-1 levels were mildly elevated in critically ill patients with COVID-19 compared to healthy controls, and that only a very small number of patients had evidence of marked glycocalyx shedding [10]. Therefore, the relationship between treatment progress and serum SDC-1 level in critically ill patients with COVID-19 has remained controversial. In this case report, we evaluated the time course of serum SDC-1 concentration in a patients with COVID-19 from admission to the ICU to the time his respiratory condition improved.

On admission to our hospital, CT of the chest showed bilateral ground glass opacities of the ARDS phenotype and serum SDC-1 level was 400.5 ng/ml. Our previous study in 78 healthy individuals receiving no treatment and with no relevant medical history or laboratory data reported a median serum SDC-1 concentration of 19.3 ng/ml [11]. Therefore, the initial serum SDC-1 level was very high in the present case, consistent with previous reports [5, 10]. Importantly, his serum SDC-1 level gradually decreased with improvement in respiratory condition, as evaluated by PaO_2_/F_I_O_2_ ratio.

In contrast, D-dimer gradually increased after hospitalization, indicating a coagulation disorder associated with COVID-19 [2]. The addition to heparin sodium was effective, and his D-dimer level peaked 6 days later. The serum SDC-1 level increased again in response to this endothelial injury due to COVID-19-associated thrombosis. Moreover, after the decrease in D-dimer, serum SDC-1 also decreased. These findings suggest that serum SDC-1 may well reflect endothelial injury associated with COVID-19.

The present patient developed aspiration pneumonia after removal of the respirator, and an increase in serum SDC-1 level was again observed. Smart et al. reported that SDC-1 and hyaluronan, a component of the glycocalyx, are significantly increased over time in patients with sepsis due to pneumonia, which is consistent with our present case [12].

The precise mechanism of shedding of SDC-1 from glycocalyx by SARS-CoV-2 is still unknown. However, Fraser et al. suggested that granzyme B and elastase 2, which are serine proteases, are elevated in ICU patients with COVID-19 relative to ICU patients without COVID-19 [13]. Elevated granzyme B and elastase 2 has been demonstrated under severe inflammatory conditions and contributes to the endothelial injury [14, 15]. Thus, these serine proteases may contribute to the degradation of glycocalyx. Indeed, several studies have reported that nafamostat, a serine protease inhibitor, is effective against COVID-19 [16, 17]. On the other hand, SDC-1 is also expressed in other organs, in addition to endothelial glycocalyx. Additionally, it is important to note that the usefulness of D-dimer in the management of patients with COVID-19 remains uncertain [18]. Moreover, we report here only a single case, yet several mutations in SARS-CoV-2 have been reported, in which the pathogenicity of the virus may differ [19]. To verify the accuracy of the change in serum SDC-1 level in COVID-19 patients, further large-scale studies are warranted.

## Data Availability

The datasets obtained and analyzed in the current study are available from the corresponding author on reasonable request.

## Abbreviations

COVID-19: Coronavirus infection 2019
ARDS: Acute respiratory syndrome
SDC-1: Syndecan-1
RT-PCR: Reverse transcription polymerase chain reaction
SARS-CoV-2: Severe acute respiratory syndrome coronavirus
CT: Computed tomography
SpO_2_: Oxygen saturation
LAMP: Loop-Mediated isothermal amplification
ICU: Intensive care unit
PaO_2_: Partial pressure of oxygen
PaCO_2_: Partial pressure of carbon dioxide
HCO_3_: Bicarbonate
SIMV: Synchronous intermittent mandatory ventilation
PEEP: Positive end-expiratory pressure
